# A realist review of the partograph: when and how does it work for labour monitoring?

**DOI:** 10.1186/s12884-016-1213-4

**Published:** 2017-01-13

**Authors:** Carol Bedwell, Karen Levin, Celia Pett, Dame Tina Lavender

**Affiliations:** 1 School of Nursing, Midwifery and Social Work, University of Manchester, Oxford Road, Manchester, M13 9PL UK; 2 Monitoring and Evaluation, Fistula Care Plus project, EngenderHealth, 440 9th Avenue, New York, NY 10001 USA; 3 Fistula Care Plus project, EngenderHealth, 440 9th Ave, 12th floor, New York, NY 10001 USA

**Keywords:** Partograph, Partogram, Labour, Context, Use, Outcomes

## Abstract

**Background:**

The partograph (or partogram) is recommended by the World Health Organisation (WHO), for monitoring labour wellbeing and progress. Concerns about limitations in the way the partograph is used in the clinical context and the potential impact on its effectiveness have led to this realist systematic review of partograph use.

**Methods:**

This review aimed to answer two key questions, 1) What is it about the partograph that works (or does not work); for whom does it work; and in what circumstances? 2) What are the essential inputs required for the partograph to work? A comprehensive search strategy encompassed key databases; including papers of varying methodologies. Papers were selected for inclusion if the focus of the paper was the partograph and related to context, mechanism or outcome. Ninety five papers were included for data synthesis. Two authors completed data extraction and synthesis.

**Results:**

The evidence synthesis relates the evidence to identified theories of health worker acceptability, health system support, effective referral systems, human resources and health worker competence, highlighting barriers and facilitators.

**Conclusions:**

This first comprehensive realist synthesis of the partograph, provides the international community of maternity clinicians with a picture of potential issues and solutions related to successful labour recording and management, which is also translatable to other monitoring approaches.

## Background

**Fig. 1:**
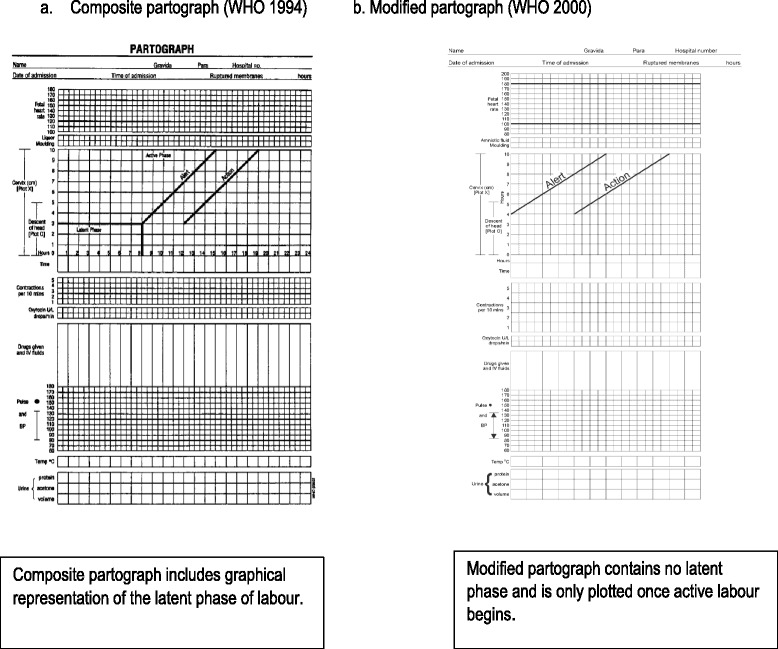
Types of Partograph. **a**. Composite partograph [18, 64]. **b**. Modified partograph [65]

The partograph (or partogram) is the most commonly used labour monitoring tool, widely supported by health professionals and recommended by the World Health Organisation (WHO) for use in active labour [1]. The purpose of the partograph is to enable health professionals to monitor wellbeing and progress in labour and provide timely intervention when required (see Fig. 1). Despite its use for over 40 years, continuing deaths from obstructed labour have led to concern that the partograph is not reaching its potential in enabling detection of deviation from the norm and timely intervention [2]. Evidence of partograph effectiveness is inconclusive; a Cochrane review suggested that overall use of the partograph did not significantly impact on a number of specified outcomes [2]. However, included trials were methodologically limited; were mainly conducted in high-income settings; and may not have included all relevant outcomes. Whilst the partograph itself may be viewed as a simple tool [3, 4], it may not be used as intended or even completed, which may suggest there are problems with the tool itself. Such problems will undoubtedly impact on outcomes [5, 6]. Barriers and facilitators to partograph use have been considered [6, 7], providing some insight into the issues, which may impact on partograph efficacy. However, whilst this increases understanding of problems facing the partograph, it does not adequately explain what is required for the tool to be clinically effective. Greater depth of understanding of the context and mechanism of partograph use is required in order to determine if and how it can reach its potential.

Literature suggests that there is widespread support for the partograph, a belief that it works and a professional will for it to succeed [4, 6, 8–10]. However, in order to understand the issues facing the partograph and its impact on outcomes, a comprehensive evaluation of the evidence is required. The partograph is used as part of an approach to labour monitoring. As such, the partograph by its nature is a complex intervention, relying on a number of factors for effective use, including interaction between a number of causal relations, behaviors and outcomes [11]. These complexities require more than a traditional review and this paper will report the findings of a review of factors which may impact on partograph efficacy using realist review methodology [12].

## Methods

A realistic review approach is appropriate for research synthesis of complex interventions such as health service delivery [12–14]. Complex interventions are embedded in health or social systems and are therefore influenced by differences in context [12]. The partograph is typical of a complex health intervention in that its use is affected by a number of factors related to design, context, implementation and management, and because it requires the active input of individuals to be effective [12]. Traditional systematic reviews are evaluative, focusing on outcome and whether or not an intervention works. The advantage of the realist review approach is that it is explanatory, allowing the researcher to explore why and how such interventions may work (or not) and in what context [12, 13].

**Fig. 2:**
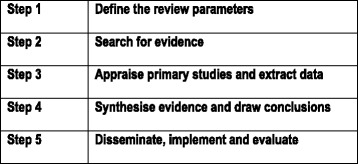
Realist review process

The review process itself consists of five steps; clarifying the scope of the review, searching for evidence, appraising primary studies and extracting data, synthesising and drawing conclusions, dissemination, implementation and evaluation (outlined in Fig. 2) which will be explained in the context of the partograph review.

### Clarifying the scope of the review

This requires identification of the review question, refining of the question and articulation of key theories to be explored. The realist review approach is described as ‘theory driven’ relying on the researcher to make explicit any presumptions of how and why the intervention works, prior to conducting the review. This also defines the overall scope of the review and provides a framework for analysis. A vital aspect in this process is input from key stakeholders, such as policy makers and experts in the field. This allows for ‘expert framing’ of the issues [12]. Key literature is also considered in determining the review theories. For this review an expert meeting ‘*Revitalizing the Partograph: Does evidence support a global call to action?’* highlighted the potential factors which may impact on correct and consistent use of the partograph [6]. Furthermore, an expert stakeholder group, consisting of global experts in the partograph, was convened. These stakeholders provided input into identification and refining of the review question and protocol development, along with review of the final report.

The review was guided by two questions

1. What is it about the partograph that works (or does not work); for whom does it work (e.g., midwives, obstetricians, women); and in what circumstances (e.g., urban/rural setting, country)?
2. What are the essential inputs required for the partograph to work?

**Fig. 3:**
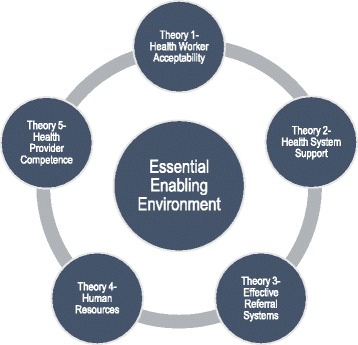
Identified theories

The review theories were developed in relation to the various aspects of the partograph as a complex intervention and the context of its use which may impact on its effectiveness. These were situated under an overarching theory of an enabling environment; that is, for the intervention to work at all the environment and context in which the intervention occurs must be supportive [15]. Five related theories were identified, consisting of: health worker acceptability, health system support, effective referral systems, human resources and health provider competence (see Fig. 3).

### Search for evidence

A comprehensive search strategy was employed to identify relevant papers for inclusion. Databases searched comprised of Medline, EMBASE, CINAHL, ProQuest, and the Cochrane database of systematic reviews. Major health advisory organisations, such as WHO were also searched for relevant policy and guidance documents. Search terms included various combinations of “partograph” OR “partogram” OR “cervicograph” OR “cervicogram” AND “labor/labour” AND “progress” AND/OR “monitor or monitoring” OR “delay” OR “tool/tools” OR “management” OR “record/recording” OR “reading” OR “chart/charting” OR “measurement” OR “length” and derivatives thereof.

**Fig. 4:**
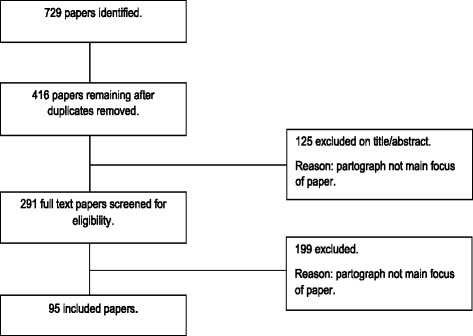
PRISMA flowchart

Purposive sampling was used to identify papers whose main focus was relevant to both the research question and the theories to be tested. Papers were included whose main focus was labour and the partograph (including partogram, cervicograph or cervicogram) and were related to the guiding theories through context, mechanism or outcome. No restrictions were applied to language, dates of publication or to the types of studies considered for inclusion. Included literature comprised of papers of various methodologies, policy and guidance documents, audits, grey literature and opinion pieces. The initial search was completed in October 2013 and repeated in October 2015. Following removal of duplicates 416 papers were screened on title and abstract by two authors. A further 291 full papers were screened, resulting in 95 papers for inclusion (see Prisma diagram, Fig. 4).

### Appraisal of evidence

Pawson [12] argues that ‘fitness for purpose’ is the most important factor in determining relevance for inclusion of evidence and rejects exclusion of papers on the basis of quality alone; the synthesis itself determines the value of the evidence. For this reason, all studies with useable data were included, regardless of quality. However, an understanding of quality is relevant for the ultimate synthesis [16]. Therefore, a quality assessment was made using the MMAT tool (version 11) [17], thus allowing for a simple review of quality for studies of varying methodologies.

### Data extraction and synthesis of the evidence

A simple data extraction tool was devised and was completed for each paper identifying aspects which related to the context, mechanism or outcome of the partograph. This provided a simple overview of the data, allowing links to be developed and related to the pre-defined theories. Pawson et al. [12] suggest that data extraction is not linear and evidence will continue to emerge during the process. This did occur with referral to the included papers continuing throughout the synthesis process as further links were developed and concepts identified. Once extracted, data were interrogated using specific questions related to each theory. This helped to clarify and synthesise the data within each individual theory.

### Results

Ninety five papers were identified for inclusion (see Fig. 4). These included primary research, reviews, guidance documents and opinion papers, in line with the realist review philosophy. For the purposes of the evidence synthesis, only primary research and review papers were included. The majority of included papers related to low-resource settings, with very few in medium or high resource settings.

The key evidence synthesis is presented below in relation to each relevant theory (Tables 1, 2, 3, 4 and 5) and will be discussed in relation to the research questions.

**Table 1:** Health worker acceptability

Question	No of studies	Evidence synthesis	Quality
Do health workers use the partograph?	*n* = 18	Wide variation in the reported routine use of the partograph in practice, from 8 to 80%. The partograph is more likely to be used in tertiary settings, by physicians and midwives. The partograph is more likely to be used in public facilities. Specific training in the partograph may increase use. There is some evidence, although limited, to suggest that experience does not have any impact on use. There is some evidence, although limited to suggest that confidence in using the partograph increases its use in practice.	**Low [25, 33, 36, 41, 48] *V low [24, 26, 28–32, 42, 43, 47, 66]
What are health workers’ attitudes towards the partograph?	*n* = 9	Evidence suggests that health workers display positive attitudes to the partograph. A positive attitude alone does not appear to increase partograph use in practice.	**Low [23, 36, 40, 41] *V low [29, 32, 42, 47, 49]
What is the impact of partograph use on clinical outcomes?	*n* = 6	Evidence from RCTs suggests there is no improvement in clinical outcomes when a partograph is used. Pre- and post-implementation studies suggest that use of the partograph may contribute to shorter labours, reduced sepsis, reduced postpartum haemorrhage, and improved fetal outcomes. There is evidence, although limited, to suggest that the partograph may improve outcomes in low-resource settings.	***Medium [18, 22, 56] *V low [19–21]
What is the impact of the partograph on quality of care?	*n* = 0	None of the included studies assessed quality of care in relation to partograph use. Data related to improved maternal outcomes post-intervention, such as fewer vaginal examinations, may indicate that women may have a better experience of labour, but there is no empirical evidence to support this.	
What is the impact of partograph use on maternal satisfaction?	*n* = 0	No studies evaluated maternal satisfaction.	
Is the partograph a useable tool?	*n* = 3	The modified partograph is easier for providers to use than the composite partograph and may improve outcomes.	***Medium [22, 23] **Low [3]

**Table 2:** Health system support

Question	No of studies	Evidence synthesis	Quality
What is the organisational commitment to partograph use?	*n* = 2	There is very little available evidence of organisational commitment. There is limited evidence of organisational commitment in high-resource settings.	**Low [23] *Very Low [29].
What is the policy and guidance related to partograph use?	*n* = 4 studies *n* = 5 guideline documents	The main guidance documents are those produced by WHO. There is a lack of available guidance at the facility level. Limited evidence suggests that available facility level guidance promotes partograph use in practice.	Guidance [64, 65, 67–70] *V low [24, 25, 30, 49]
Is the partograph available?	*n* = 8	There is a lack of availability of the partograph in some settings, particularly health centres.	**Low [25, 33, 38, 43, 48] *V low [29, 30, 47]
Is there support for partograph use in terms of resource provision?	*n* = 2	Equipment required for partograph completion may not be available; for example sphygmomanometers, thermometers and fetoscopes.	*V low [29, 31]
How can the partograph be implemented effectively?	*n* = 2, plus 1 audit	There is little evidence to determine the most effective method of partograph implementation. Pre-implementation training and post-implementation audit and feedback may have a positive impact on accuracy and frequency of partograph completion.	***Medium [18] *V low [20, 50]

**Table 3:** Effective referral systems

Question	No of studies	Evidence synthesis	Quality
Which methods of working ensure effective referral?	*n* = 2	There is confusion between healthcare worker roles, particularly between midwives and physicians, which may impact on the effectiveness of referral. The partograph is not always used as a communication tool between health workers at handover of care or referral. Partograph findings are not always acted upon.	*V low [29, 32]
What are the issues or barriers related to effective referral?	*n* = 10	The partograph is a trigger for referral. However, there is some inconsistency in referrals based on partograph findings. It is unclear if referrals made as a result of partograph use are appropriate. There was little evidence of additional barriers to transfer, e.g., transport, cost etc.	**Low [23, 36, 38] *V low [20, 28, 29, 31, 32, 37, 39]

**Table 4:** Human resources

Question	No of studies	Evidence synthesis	Quality
Is there sufficient availability of personnel to enable effective partograph use?	*n* = 9	Staff shortages and a heavy workload appear to negatively impact partograph use. Some health workers find the partograph time-consuming to complete. The was some evidence to suggest the partograph is completed in retrospect The partograph can successfully be completed by non-professional cadres.	**Low [23, 25, 33, 43, 48] *Low [29, 30, 32, 49]
What supervision and mentoring of staff is required?	*n* = 3, plus 1 audit	Supervision may have a positive influence on partograph completion and use. Audit and feedback of findings to staff may improve completion rates and quality of completion.	**Low [23] *V low [28, 37, 50]

**Table 5:** Health worker competence

Question	No of studies	Evidence synthesis	Quality
What is health workers knowledge of assessment using the partograph?	*n* = 10	Knowledge of assessment using the partograph is generally poor, particularly when to start the partograph, the plotting of normal labour and the function of the action and alert lines. Knowledge is better in health workers with professional qualifications and those in tertiary settings. There is a little available evidence of health workers’ understanding of the partograph as a tool to aid decision making.	**Low [33, 36, 41, 43, 48] *V low [26, 30, 32, 42, 71]
Do education, training and experience impact on knowledge of the partograph?	*n* = 5	Professional education and/or training in partograph use improve knowledge of the partograph. There does not appear to be a link between length of experience in using the partograph and knowledge of the partograph.	**Low [41, 43, 48] *V low [30, 32]
What is the level of competence in partograph completion?	*n* = 11	The overall standard of partograph recording is poor and frequently not in accordance with WHO or other guidance. Particular aspects of the partograph are more likely to be completed; these are cervical dilatation, fetal heart rate, and condition of the neonate. Maternal observations are least likely to be completed well.	**Low [25, 33, 36] *V low [24, 26–31, 66]
Do training interventions increase knowledge and use of the partograph?	*n* = 8	Training interventions do appear to improve knowledge and use of the partograph. Individualised training sessions and self-directed training (e.g., CD-ROM or maternal care manual) are most effective in increasing knowledge (in the included studies). Health workers desire training in partograph use, even if they have already received training.	***Medium [56] **Low [54] *V low [34, 35, 37, 53, 72]

## Discussion

This review aimed to answer two key guiding questions, which will be discussed below.

### What is it about the partograph that works (or does not work); for whom does it work (e.g., midwives, obstetricians, women); and in what circumstances (e.g., urban/rural setting, country)?

#### What is it about the partograph that works?

A Cochrane review of the partograph suggests that evidence to support improvement of clinical outcomes is limited^2^. However, evidence from other studies indicates that partograph use may contribute to shorter labours and some improvement in maternal and fetal outcomes [18–21]. Health care workers found the modified partograph the most user-friendly version [3, 22, 23], with the latent phase of the composite partograph considered difficult to complete. The modified partograph also appears to improve outcomes including reduced caesarean section rate, augmentation of labour and admission to neonatal unit when compared to the composite partograph in low-resource settings [23].

Overall completion of the partograph (to pre-defined standards) is poor, which is likely to impact on the utility of the tool in clinical practice. The sections of the partograph which are most likely to be completed are those relating to progress (cervical dilatation) and fetal wellbeing (fetal heart rate) [24–27]. Those that were poorly completed related to maternal wellbeing [24–28]. However, this may reflect ease of use in completing particular sections, availability of equipment or participants understanding of the partograph, rather than the tool itself [3, 29–31]. Whilst some view the partograph as difficult or time consuming to complete [30, 32, 33], there is evidence that other, non-professional, cadres of staff can complete the partograph effectively [34, 35].

The partograph does appear to work as a trigger for referral and transfer [20, 28, 31, 32, 36–38], one of its primary purposes. However, evidence related to other types of decision-making, for example augmentation of labour, based on partograph findings is limited and there is some suggestion that partograph findings may not always be acted upon [29]. The success of the partograph as a communication tool at handover of care is limited [23, 29] and women who are transferred to tertiary units are not always sent with the partograph commenced at the referring facility [39].

#### For whom does it work?

In terms of improved outcomes for women and newborns, there is conflicting evidence as to whether the partograph works, although studies in low-resource settings suggest that it may have positive impact [18–21]. There is no evidence that partograph use is detrimental to outcomes [2]. Whilst there is no data related to maternal satisfaction or quality of care when the partograph is used, suggestions of fewer vaginal examinations, reduced length of labour and referral may indicate that women are receiving more appropriate treatment and intervention [18–21, 37].

Midwives appear to be satisfied with the partograph as a usable tool for monitoring labour [18, 40]. Positive attitudes towards the partograph are displayed by both midwives and doctors, but less so by other cadres of health care worker who also use the partograph [32, 36, 40–42]. It is clear that positive attitudes alone do not translate into partograph use in practice. This may be for a number of reasons such as availability, time, workload and organisational culture [23, 29–32, 43].

One advantage of partograph use is that it enables health workers to take individual responsibility for labour management within their own sphere of practice [44]. However, confusion over roles and responsibilities for the partograph existed in some settings [29, 32]. Such role confusion may be an indication of general poor multi-disciplinary working and communication, which may ultimately impact on decision-making and outcomes [45]. Similarly, training in partograph use can be an issue where either supervisors are not trained [29], or training is not provided to those who are using the partograph on a daily basis [25].

Although the partograph is considered a ‘cheap’ intervention at less than 10 US cents per paper version [46], there was no evidence of evaluation of the cost-effectiveness of the partograph in the included literature.

#### In what circumstances?

The partograph appears widely accepted, but in practice, its use varies considerably. Barriers to use include poor availability of partograph or equipment to complete it, high workloads, poor staffing levels, duplication of records, lack of available policy or guidance and limited knowledge and understanding of the partograph [29, 30, 32, 33, 36, 41–43, 47, 48].

The partograph is most likely to be used in urban, tertiary facilities and by professionally qualified staff or those trained in partograph use. This is perhaps not surprising as tertiary settings are most likely to have funding for training and also employ a greater proportion of qualified staff. The partograph is also more likely to be used in public facilities. The availability of policy or guidance at facility level also appeared to have a positive impact on partograph use [30, 49]. Much more limited evidence was available for consistent and accurate partograph use in the long term. Studies reintroducing the partograph or retraining staff suggest problems with maintaining consistent partograph use after introduction. Ongoing supervision and support in practice is likely to improve partograph use, but current evidence is limited to that within study settings [37]. Similarly, repeated audit and feedback contribute to ongoing regular and accurate use in practice [50].

### What are the essential inputs required for the partograph to work?

Many of the issues relating to partograph use arise from the difficulties of putting it into practice effectively. There is limited description of implementation strategies in the reviewed literature. The poor levels of compliance and the implication that the partograph is not embedded in routine care are suggestive that the partograph has not been ‘normalised’ into care processes [11]. As such there appears to be a lack of overall commitment, resulting in varied (at best) acceptability and use of the partograph in clinical practice. For the partograph to work, it needs to be acceptable to health care workers who provide care to women in labour. Currently, although the majority of health care workers have positive attitudes towards the partograph, a number of factors need to be overcome to ensure an enabling environment facilitating consistent and effective use of the partograph in practice. These include clear health system support and commitment, availability of resources, competence in use and monitoring and evaluation of the partograph in practice.

Health system support and commitment is vital in promoting a positive culture for partograph use [51]. The role of the health system itself was not addressed in the literature relating to the partograph, yet the need to strengthen such systems is well acknowledged in the wider literature [51, 52]. Current evidence suggests a culture where the partograph is not central to care and where adequate supervision is lacking. Positive validation of the partograph from leaders, managers and supervisors is required; reinforcement by guidance, audit and evaluation of partograph use will assign value to the tool. ‘Buy in’ by supervisors and leaders is important, as they are most likely to be influential in promoting a positive environment for partograph use. Furthermore, supervisors in the clinical setting can provide guidance and confirm clinical decision making based on partograph findings. Multidisciplinary working is also crucial in effective use of the partograph and is more likely to be sustained and effective with health system support [45]. Facility level guidance should be available and accessible to health care workers providing care to women in labour. This should comprise both guidance on interventions and the responsibilities of individuals in the care setting. Support in terms of provision of resources, such as the partograph itself and equipment required for completion is vital at a basic level to ensure consistent use but which is currently lacking.

Current studies indicate poor health worker competence in partograph use. Training in partograph use does increase knowledge and completion of the partograph in practice [30, 32, 34, 35, 37, 41, 43, 53–56] and is essential in any facility in which the partograph is used. Frequent and high turnover of staff indicate that this is required on a regular basis. Furthermore, in many of the included studies, staff who had already been trained requested further training, indicating that consistent reinforcement is necessary to develop and maintain skills [23, 25, 30–32, 36, 43]. Although individualised training has been demonstrated to work in improving partograph knowledge [55, 56], use of multidisciplinary training strategies may improve understanding of roles and promote team working [57]. Practical training methods can also enhance learning and may improve patient outcomes [58–60]. All staff providing care for women in labour should be trained and regularly updated in partograph use. Such training needs to follow established effective training methods, such as multidisciplinary training models [60]. It is essential that the content of such training includes both completion and decision making skills, such as when to start the partograph, when to take action and appropriate referral pathways.

Health worker training aids the development of competence in and increases partograph use in the short term, but long-term maintenance is essential. Indications that organisational culture can negatively affect use [23, 29], may require consideration of behavior change strategies for both health workers and supervisors, which are suitable for the setting [61]. In order to promote long-term partograph use, ongoing audit, evaluation and feedback is necessary. Audit and feedback, provided by a supervisor, can lead to improved performance in terms of professional practice and has also been found to improve partograph use [50, 62]. Such a strategy will enable learning, demonstrate continued health system commitment to the partograph and provide much needed data in relation to outcomes. One issue with the partograph in current use is the failure to evaluate the tool at facility level in terms of outcomes. This is vital in determining the level of impact partograph use has on care provision and referrals as well as on specific labour outcomes. Furthermore, if health workers and organisations can observe positive outcomes from partograph use it is more likely to become embedded into practice.

### Limitations

There were some limitations to this review. Few included studies considered more than one aspect of partograph use, such as mechanism of use, and this was not related to either context or outcomes; although understanding and inferences can be drawn from the studies that are available. This limitation is accepted as part of the realist review process [12]. Furthermore, the overall quality of evidence was generally low or very low. Although quality was not an inclusion criterion for papers, it must be taken into consideration in interpreting the findings. It is also possible that other factors, which fall outside of the scope of the review, may impact on partograph use, such as health workers understanding of the physiology of labour. Finally, whilst some general recommendations can be made, it is important to acknowledge that the scope of the realist review process is to provide suggestions and to add depth to established theories, rather than to provide universal recommendations that may be expected to work in all contexts [12].

### Recommendations

A number of recommendations can be made as a result of this review:

- The modified partograph is preferable to the composite partograph in terms of ‘user friendliness’.
- The partograph and equipment required to complete it need to be available.
- The partograph should be the main labour record, reducing unnecessary duplication of documentation.
- There should be clear policy/guidance available at facility level for healthcare workers’ reference.
- Effective supervision by healthcare workers/managers with training and clinical experience in partograph use is necessary for sustaining successful implementation.
- Regular training and updating should be provided for all healthcare workers using the partograph, using proven effective training techniques, e.g., multi-disciplinary, practical/clinical application. Training should include understanding of when to commence the partograph, decision making based on findings and understanding of role.
- Monitoring and audit of the partograph in practice, including completion, decision making and referral and outcomes, is recommended.

## Conclusion

This review is the first comprehensive realist synthesis of the complex issues surrounding partograph use. The partograph was introduced at a time when evaluation of new interventions was not commonplace. Subsequent studies have considered various aspects of partograph use and outcomes, but none have fully encompassed the challenges of implementing and evaluating such a complex intervention. Clinically, although the partograph appears to be accepted, there is evidence that it is not being used as anticipated in practice, hence it is failing to reach its potential in improving outcomes. This review provides clinicians with a comprehensive overview of the potential challenges and solutions related to labour recording and management. Clinicians can now take these findings and assess their transferability to their own units, taking into consideration their own context and processes. These findings also provide important considerations which may have application to the development of new labour monitoring tools, such as the simplified effective labour monitoring-to-action tool [63].

In the case of the partograph, this review has revealed the urgent need definitive trial in both low and high-resource settings, to include not only clinical outcomes, but also quality of care, client satisfaction, health economics, impact on methods of working; along with a comprehensive implementation and evaluation strategy. This is a vital step in determining the effectiveness and future role of the partograph in practice.

## Abbreviations

WHO: World Health Organisation
